# The Roles of Mesenchymal Stem Cells in Gastrointestinal Cancers

**DOI:** 10.3389/fimmu.2022.844001

**Published:** 2022-02-24

**Authors:** Ze Xiang, Menglu Hua, Zhou Hao, Huang Biao, Chaojie Zhu, Guanghua Zhai, Jian Wu

**Affiliations:** ^1^ School of Medicine, Zhejiang University, Hangzhou, China; ^2^ Affiliated Hangzhou Chest Hospital, Zhejiang University School of Medicine, Hangzhou, China; ^3^ College of Life Sciences and Medicine, Zhejiang Sci-Tech University, Hangzhou, China; ^4^ Department of Clinical Laboratory, The Affiliated Suzhou Hospital of Nanjing Medical University, Suzhou Municipal Hospital, Gusu School, Nanjing Medical University, Suzhou, China

**Keywords:** mesenchymal stem cells, gastrointestinal cancers, bilateral roles, mediating mechanism, therapeutic targets

## Abstract

Mesenchymal stem cells (MSCs) were reported to have strong immunomodulatory ability, and inhibit the proliferation of T cells and their immune response through cell-to-cell interactions and the generation of cytokines. With high differentiation potential and self-renewal ability, MSCs are considered to function in alleviating inflammatory responses, promoting tissue regeneration and inhibiting tissue fibrosis formation. As the most common malignancies, gastrointestinal (GI) cancers have high incidence and mortality. The accurate diagnosis, exact prognosis and treatment of GI cancers have always been a hot topic. Therefore, the potential applications of MSCs in terms of GI cancers are receiving more and more attention. Recently, there is increasing evidence that MSCs may serve as a key point in the growth, metastasis, inhibition, treatment and prognosis of GI cancers. In this review, we summarized the roles of MSCs in GI cancers, mainly focusing on esophageal cancer (EC), gastric cancer (GC), liver cancer (LC), colorectal cancer (CRC) and pancreatic cancer. Besides, we proposed MSCs as potential targets and treatment strategies for the effective treatment of GI cancers, which may provide better guidance for the clinical treatment of GI cancers.

## Introduction

Mesenchymal stem cells (MSCs) are pluripotent stem cells with high differentiation potential and self-renewal ability, which are derived from the mesoderm and ectoderm in early development (1). MSCs were originally found in the bone marrow and could be induced to differentiate into multiple cell types, such as osteoblasts, chondrocytes, adipocytes and endothelial cells (2). It was reported that MSCs could secrete more than 100 cytokines, functioning as an immunomodulator, Moreover, they could also accurately migrate to damaged tissues and organs for tissue repair, which would play a key role in alleviating inflammatory responses, promoting tissue regeneration and inhibiting tissue fibrosis formation (3). Stem cell therapy has always been emphasized (4, 5).

With the high incidence and mortality, gastrointestinal (GI) cancers are considered as the most common malignancies, mainly including esophageal cancer (EC), gastric cancer (GC), liver cancer (LC), colorectal cancer (CRC) and pancreatic cancer (6–8). In recent years, an increasing number of studies have revealed the important pro-tumor and anti-tumor roles of MSCs in GI cancers. It was shown that MSCs could promote angiogenesis by secreting pro-angiogenic factors. MCSs could not only act directly on GI cancer cells through signaling pathway, but also indirectly act through secreting exosomes, thereby promoting the metastatic invasion of tumor cells and inhibiting the growth of tumor cells, which may provide a potential target for the treatment of GI cancers. In addition, MSCs could also protect cancer cells from the cytotoxic effects of anti-cancer drugs and thus promote drug resistance in cancer cells. Due to the strong regenerative potential and immunomodulatory ability, the roles of MSCs are widely focused on in terms of GI cancers.

Overall, MSCs are of great significance in the pathogenesis, disease progression, treatment and prognosis of different GI cancers. This review summarized the mechanism and treatment of MSCs in GI cancers and proposed MSCs as potential targets and treatment strategies for the effective treatment of GI cancers, aiming to provide new ideas for the clinical treatment of GI cancers.

## Roles of Mesenchymal Stem Cells in Esophageal Cancer

EC is a common gastrointestinal tumor, which often occurs in the innermost layer of the esophageal tissue and infiltrates outward (9, 10). There exist two common types, adenocarcinoma and squamous cell carcinoma included. The former may be associated with obesity, while the latter is related to heavy alcohol consumption and drug use. Approximately 300,000 people die from EC each year in the world (11). Of note, it was revealed that MSCs could serve as a key point in EC in several studies.

EC could be inhibited by various MSCs *via* different ways. Through the analysis of transcriptome changes after cell fusion, Wang et al. found that the fusion of human umbilical cord MSCs (hUCMSCs) and EC9706 cells may inhibit the growth of EC cells, mainly through inducing pro-apoptotic signaling and DUSP6 negative feedback inhibition mechanisms (12). Similarly, it was also found that the fusion of hUCMSCs and EC cells could induce apoptosis and benign trans-differentiation, thus inhibiting the tumorigenicity of EC cells (13). Kumar et al. confirmed that chemerin secreted by EC myofibroblasts could recruit bone marrow-derived MSCs, thereby delaying tumor progression (14). Through the adenovirus-mediated TRAIL gene transduction, Li et al. demonstrated that MSCs with TRAIL gene could inhibit the proliferation of EC cells and induce apoptosis. This conclusion was further verified in mouse models, which may be of potential value for improving the treatment of EC (15).

Certain MSCs may promote the growth and progression of EC. The role of human MSCs (hMSCs) on EC were controversial. Tian et al. discovered that hMSCs could inhibit the proliferation and invasion of EC cells *in vitro*. Nevertheless, in animal models, hMSCs were confirmed to enhance tumor formation and growth to the contrary (16). Moreover, Yang et al. showed that hUCMSCs could promote the formation of EC. MSCs was also proved to be recruited by EC cells, thereby promoting EC cell migration and invasion (17). Therefore, the exploitation of hMSCs and hUCMSCs in new therapeutic strategies should be cautious.

MSC-derived exosomes could deliver specific microRNAs (miRs) to influence the physiological functions of EC cells. Exosome miRs derived from bone marrow MSCs (BMSCs) was considered as a promising cancer treatment strategy. Deng et al. found that BMSC-derived exosome miR-19b-3p could promote EC progression by targeting SOCS1 (18). In addition, it was demonstrated that hUCMSC-derived exosomes could transmit miR-375 to inhibit enabled homolog expression, thus inhibiting the occurrence and progression of esophageal squamous cell carcinoma (19).

In general, MSCs and their derivatives play a key role in the occurrence, progression and possible treatment of EC. However, the relevant studies are insufficient, and its involving mechanism has not been fully explored. Thus, more studies are needed.

## Roles of Mesenchymal Stem Cells in Gastric Cancer

GC is a malignant tumor that originates from the epithelium of the gastric mucosa. Most GCs are adenocarcinomas with no obvious symptoms in the early stages, which are easy to ignore (20). GC is the leading cause of cancer-related deaths worldwide. Although the use of surgery combined with chemotherapy and radiotherapy has improved, the prognosis of advanced GC is still poor (21). In recent years, more and more studies showed that MSCs are closely related to the occurrence and development of GC, which may provide a guide for the exact diagnosis and accurate diagnosis of GC (**Table 1**).

**Table 1:** The roles of different MSCs in gastric cancer and their inducing mechanisms.

Author	MSCs and their derivatives	Inducing ways	Role
Zhang et al. (22)	MSCs activated by macrophages	Obtaining pro-inflammatory phenotypes.	Enhancing the oncogenic transformation.
Yang et al. (23)	MSCs activated by macrophages	Through NF-κB-dependent manner.	Promoting the occurrence of GC.
Chen et al. (24)	BMSCs	Regulating c-Myc.	Promoting the growth of GC.
Li et al. (25)	GC-MSCs	Secreting IL-8.	Promoting the progression of GC.
Gu et al. (26)	Exosomes derived from hMSCs	Activating the Akt pathway.	Improving the migration of GC cells.
Wang et al. (27)	GC-MSCs	Transferring exosome miRNAs to GC cells.	Promoting the progression of GC.
Ikeda et al. (28)	MSC-derived CXCL16	Via the expression of Ror1 mediated by STAT3.	Promoting the progression of GC cells.
Takiguchi et al. (29)	MSCs	Activating the CXCL16-CXCR6 axis.	Promoting the proliferation of GC.
Chen et al. (30)	GC-MSCs	Up-regulating the expression of HK2 through G6PD-NF-κB-HGF signal	Promoting the proliferation and metastasis of GC cells.
Wang et al. (31)	GC-MSCs	Disrupting the balance of Treg/Th17.	Influencing the progression of GC.
Guo et al. (32)	GC-MSCs	Inhibiting NK cell function through mTOR signal	Promoting the tumor growth.
Huang et al. (33)	GC-MSCs	Secreting platelet-derived growth factor-DD.	Promoting the progression of GC cells.
Yin et al. (34)	GC-MSCs	Inactivating the Wnt/β-catenin signal.	Limiting the increased activity of GC cells and reversing the EMT process induced by GC-MSCs
Zhu et al. (35)	hUCMSCs carrying LIGHT gene	–	Posing killing effects on GC.
Ma et al. (36)	hUCMSCs carrying LIGHT gene	–	Posing killing effects on GC.
Zhao et al. (37)	Human adipose MSCs	–	Inhibiting the proliferation of HGC-27 cells in GC and inducing the apoptosis.
Wang et al. (38)	hUCMSCs pretreated with IL-6	–	Inhibiting cell proliferation and inducing apoptosis.
Pan et al. (39)	GC-MSCs	Knocking down YAP signaling	Inhibiting the promotion effect of GC-MSCs on GC growth.
Ruan et al. (40)	MSCs labeled with fluorescent magnetic nanoparticles	–	Targeting GC cells *in vivo*
Author	MSCs and their derivatives	Inducing ways	Role
Sun et al. (41)	GC-MSCs expressing PD-L1	–	Resulting in GC cells becoming resistant to chemotherapy.
He et al. (42)	MSC-regulated lncRNA MACC1-AS1	Through fatty acid oxidation.	Promoting chemotherapy resistance in GC cells.
Wu et al. (43)	MSC-induced lncRNAHCP5	Driving fatty acid oxidation through the miR-3619-5p/AMPK/PGC1α/CEBPB axis.	Promoting chemical resistance to GC.
Weber et al. (44)	hMSC-derived exosomes	–	Enhancing GC resistance.
Ji et al. (45)	BMSCs	Regulating the PI3K/AKT pathway.	Increasing the drug resistance of GC cells.
Zhou et al. (46)	Oxygen-carrying MSCs	–	Enhancing the effect of chemotherapy for GC *in vitro*.
Sun et al. (47)	IL-8 from GC-MSCs	Inducing PD-L1 expression in GC cells through STAT3/mTOR-c-Myc signaling axis.	Overcoming PD-L1-induced immune escape in GC cells.
Xu et al. (48)	GC-MSCs	CD4+ T cell stimulation.	Enhancing the growth of GC cells.
Numakura et al. (49)	CD73, CD90 and CD105	–	Participating in the progression of GC.
Carbone et al. (50)	BMSC-derived exosome miR-221	–	Serving as an important detection indicator of GC.

MSCs, Mesenchymal stem cells; GC, Gastric cancer; BMSCs, Bone marrow mesenchymal stem cells; hMSCs: Human mesenchymal stem cells hUCMSCs: Human umbilical cord mesenchymal stem cells; PD-L1, Programmed cell death ligand 1; miRs, MicroRNAs.

MSCs could promote the growth and metastasis of GC in various ways. It was revealed that MSCs activated by macrophages could obtain pro-inflammatory phenotypes to reshape the inflammatory microenvironment, thereby enhancing the oncogenic transformation of gastric epithelial cells (22). Similarly, Yang et al. demonstrated that MSCs activated by macrophages could acquire a pro-inflammatory phenotype and promote the occurrence of GC in the NF-κB-dependent manner (23). It was also suggested that BMSCs could promote the growth of GC by regulating c-Myc (24). By secreting a large amount of IL-8, Li et al. proved that GC-MSCs could effectively promote the growth and progression of GC (25). Moreover, exosomes derived from hMSCs were confirmed to promote the growth and migration of GC cells through activating the Akt pathway (26). Interestingly, MSCs derived from GC tissues were also demonstrated to promote the progression of GC by transferring exosome miRNAs to GC cells (27). Ikeda et al. found that CXCL16 derived from MSCs could also promote the progression of GC cells through the expression of Ror1 mediated by STAT3 (28). Similarly, Takiguchi et al. demonstrated that the Wnt5a-Ror2 signaling pathway in MSCs could promote the proliferation of GC by activating the CXCL16-CXCR6 axis (29). The expression of HK2GC could be up-regulated by MSCs through G6PD-NF-κB-HGF signal, thereby promoting the proliferation and metastasis of GC cells (30). In addition, GC-MSCs could damage the anti-tumor immune response of peripheral blood mononuclear cells by disrupting the balance of Treg/Th17, thereby affecting the progression of GC (31). Guo et al. also revealed that GC-MSCs could inhibit NK cell function through mTOR signal, thereby promoting tumor growth (32). Platelet-derived growth factor-DD secreted by GC-MSCs could promote the progression of GC cells both *in vitro* and *in vivo* (33). Of note, Yin et al. concluded that resveratrol can limit the increased activity of GC cells and reverse the epithelial mesenchymal transformation (EMT) process induced by GC-MSCs through inactivating the Wnt/β-catenin signal (34).

There was increasing evidence that MSCs may pose an inhibitory effect on GC. Several studies showed that hUCMSCs carrying the LIGHT gene would have killing effects on GC (35, 36). Human adipose MSCs could inhibit the proliferation of HGC-27 cells in GC and induce their apoptosis (37). Human umbilical cord-derived MSCs pretreated with IL-6 could significantly eliminate the ability of hUCMSCs to promote the proliferation and migration of gastric epithelial cells (38). Pan et al. found that YAP signal transduction in GC-derived MSCs is critical to the promotion of GC cells. They also found that by knocking down YAP signaling, the growth, migration and invasion of GC-MSCs could be inhibited, then inhibiting the promotion effect of GC-MSCs on GC growth (39).

The applications of MSCs are critical in the treatment of GC. It was suggested that MSCs labeled with fluorescent magnetic nanoparticles could target GC cells *in vivo*, which own great application potential in imaging, diagnosis and hyperthermia treatment of early GC (40). Chemotherapy is the treatment of choice for patients with advanced GC. Nevertheless, chemotherapy resistance is still a major barrier to effective treatment of GC. It was shown a strong association between MSCs and the resistance of GC. Sun et al. found that GC-MSCs expressing programmed cell death ligand 1 (PD-L1) could enhance the cancer stem cell-like properties of GC cells, resulting in GC cells resistant to chemotherapy (41). He et al. found that MSC-regulated lncRNA MACC1-AS1 could promote chemotherapy resistance in GC cells through fatty acid oxidation, indicating that the combination of fatty acid oxidation inhibitors and chemotherapy may be promising to overcome chemotherapy resistance (42). Besides, Chemical resistance to GC could also be promoted by MSC-induced lncRNAHCP5 through the miR-3619-5p/AMPK/PGC1α/CEBPB axis to promote (43). Exosomes derived from hMSCs could similarly enhance GC resistance (44). Through regulating the PI3K/AKT pathway, BMSCs were proved to increase the drug resistance of GC cells, indicating that targeting this pathway may help improve the efficacy of chemotherapy in GC (45). In addition to chemotherapy tolerance, hypoxia also serves as an important factor in chemotherapy failure for most solid tumor types, especially for GC. Zhou et al. found that oxygen-carrying MSCs could enhance the effect of chemotherapy for GC *in vitro*. The application of MSCs as a carrier to supply oxygen to solid tumors could improve the hypoxia of tumor tissues and increase the effect of chemotherapy on tumor cells (46). Additionally, MSCs also matters in the immunotherapy of GC. Sun et al. found that IL-8 from GC-MSCs could induce PD-L1 expression in GC cells through STAT3/mTOR-c-Myc signaling axis, thereby causing GC cells to develop resistance to the cytotoxicity of CD8+ T cells. Therefore, blocking IL-8 derived from GC-MSCs could overcome PD-L1-induced immune escape in GC cells (47). It was revealed that CD4+ T cells could stimulate GC-MSC and change the immune phenotype of GC-MSC, then enhancing the growth of GC cells, providing new insight into the immunotherapy of GC (48).

The prognosis of GC is also closely associated with MSCs. Satoe et al. discovered that spindle-shaped GC stromal cells expressing some specific MSC markers, such as CD73, CD90, and CD105, could participate in the progression of GC. Among them, CD105-positive cells may be closely related to poor prognosis (49). The high expression of exosome miR-221 derived from BMSCs in peripheral blood is positively correlated with the poor clinical prognosis of GC, especially at the stage of tumor, lymph node and metastasis. Therefore, the expression of miR-221 in peripheral blood was considered to serve as an important detection indicator of GC, which suggested that the use of miR-221 inhibitors with excellent inhibitory effects in exosomes may be a potential strategy for the treatment of GC in the future (50).

The roles of MSCs in the growth, metastasis, inhibition, treatment and prognosis of GC have received more and more attention. Recently, many studies focused on the mechanism of MSCs acting on GC, which may provide potential targets and therapeutic strategies for the treatment of GC. However, the practical clinical applications of MSCs to GC is still relatively less, and more efforts should be made to explore the possibility of the clinical applications of MSCs in GC.

## Roles of Mesenchymal Stem Cells in Liver Cancer

LC is a malignant tumor that occurs in the liver and can be divided into primary and secondary LC. Primary LC occurs in the epithelial or mesenchymal tissue of the liver and is extremely harmful, among which hepatocellular carcinoma (HCC) is the most common (51). Secondary LC refers to the invasion of liver by malignant tumors originating from multiple organs throughout the body. There are almost 840,000 new cases and 780,000 deaths associated with LC worldwide each year, with the mortality rate of 8.2% (52, 53). The biomarkers in liver cancer have always been a hot topic (54). Recently, the treatment of LC by MSCs has also attracted much attention (**Table 2**). Qiao et al. found that MSCs could inhibit the malignant phenotype of the HepG2 human HCC cell lines, which provided a new approach and experimental basis for cancer treatment (55). The important role of MSCs in the metastasis, invasion, and inhibition and treatment of LC have been confirmed, so the role of MSC in LC should be paid more attention.

**Table 2:** The roles of different MSCs in liver cancer and their inducing mechanisms.

Author	MSCs and their derivatives	Inducing ways	Role
Qiao et al. (55)	MSCs	–	Inhibiting the malignant phenotype of the HepG2 HCC cell lines.
Wang et al. (56)	HCC-associated MSCs	Through the DNM3OS/KDM6B/TIAM1 axis.	Promoting HCC metastasis.
Liu et al. (57)	UCMSCs	Via the TGF-β-induced EMT.	enhance tumor cell metastasis
Jing et al. (58)	MSCs in the inflammatory microenvironment	Via the TGF-β-induced EMT.	Promoting HCC metastasis.
Yan et al. (59)	LC-MSCs	Secreting S100A4.	Leading to an increase in the aggressiveness of LC tumors.
Mi et al. (60)	BMSCs	Secreting IL-6.	Promoting HCC metastasis.
Pelagalli et al. (61)	BMSCs	Through the AQP1 participation.	Causing HCC cell migration and invasion.
Chen et al. (62)	hMSCs	By the MAPK pathway.	Promoting the tumor growth.
Teshima et al. (63)	Soluble factors in adipose tissue MSCs	–	Contributing to the proliferation and invasion of canine LC cells.
Cai et al. (64)	NK4 modified MSCs	–	Inhibiting the growth and migration of MHCC-97H cells and tumor angiogenesis.
Byun et al. (65)	Adipose tissue-derived MSCs	By the IFN-β-mediated JAK/STAT1 pathways.	Inhibiting the growth of Huh7 HCC cells.
Tang et al. (66)	UCMSCs	Down-regulating AFP, Bcl-2 and Survivin.	Inhibiting the growth of HepG2 cells and promote their apoptosis.
Su et al. (67)	BMSCs	Reducing the levels of Notch1 expression.	Inhibiting the progression of LC cells.
Xie et al. (68)	INF-β modified BMSCs	Secreting high levels of IFN-β and inhibiting the AKT/FOXO3a pathway.	Inhibiting HCC.
Wu et al. (69)	MSC-HNF4α	Down-regulating the Wnt/β-catenin signaling pathway.	Reducing the growth and metastasis of LC cells.
Wang et al. (70)	Co-expression of IFN-γ and IL-10 in BMSCs	Modulating cell cycle regulators and MAPK pathways.	Inhibiting HCC.
Author	MSCs and their derivatives	Inducing ways	Role
Hou et al. (71)	MSCs	Inhibiting the expression of Wnt signaling pathway-related factors.	Inhibiting the proliferation of HepG2 cells and promoting their apoptosis.
Abdel et al. (72)	MSCs	Inhibiting the expression of Wnt signaling pathway-related factors.	Inhibiting the proliferation of HepG2 cells and promoting their apoptosis.
Deng et al. (73)	miR-20a-3p derived from MSC- extracellular vesicles	Targeting c-FLIP and increasing the levels of TRAIL.	Promoting the TRAIL-related cell apoptosis.
Li et al. (74)	BMSC-derived exosome miR-338-3p	Targeting ETS1.	Inhibiting the progression of HCC.
Seyhoun et al. (75)	MSCs	Combining sorafini with MSCs.	Posing a synergistic anti-tumor effect on LC cells.
Seyhoun et al. (76)	MSCs	Combining sorafini with MSCs.	Posing a synergistic anti-tumor effect on LC cells.
Groth et al. (77)	Porcine MSCs	–	Functioning similar to hMSCs.
Deng et al. (78)	Rat MSCs	Providing a stable source of stTRAIL.	Suitable for recurrence of HCC in patients after receiving radiofrequency ablation.
Yuan et al. (79)	hUCMSCs	Replicating adenoviruses to specifically eliminate HCC cells.	Removing postoperative residues of HCC and avoid the metastasis.
Li et al. (80)	MSCs	–	Causing the recurrence of HCC.

MSCs, Mesenchymal stem cells; LC, Liver cancer; HCC, Hepatocellular carcinoma; UCMSCs, Umbilical cord mesenchymal stem cells; EMT, Epithelial mesenchymal transformation; BMSCs, Bone marrow mesenchymal stem cells; hMSCs, Human mesenchymal stem cells; hUCMSCs, Human umbilical cord mesenchymal stem cells; miRs, MicroRNAs; stTRAIL, secretable form of TRAIL.

MSCs could influence the metastasis and invasion ability of LC cells. To explore the mechanism between MSC and HCC cells, Wang et al. discovered that HCC-associated MSCs can promote HCC metastasis through the DNM3OS/KDM6B/TIAM1 axis (56). Since TGF-β could induce EMT in HCC cells, Liu et al. found that umbilical cord MSCs (UCMSCs) could significantly enhance tumor cell metastasis (57). Similarly, MSCs in the inflammatory microenvironment were also confirmed to promote HCC metastasis through TGF-β-induced EMT (58). S100A4 secreted by LC-MSCs could increase the expression of miR-155, which would promote the expression of matrix metalloproteinase 9, leading to an increase in the aggressiveness of LC (59). BMSCs were also showed to secrete IL-6 to promote HCC metastasis (60). After recruiting human BMSCs into the tumor microenvironment, Pelagalli et al. demonstrated that BMSCs may cause HCC migration and invasion with the participation of AQP1 (61). Chen et al. confirmed that hMSCs would promote tumor growth through the MAPK pathway, and they found that the over-expressed integrin α5 promotes hMSC-induced HCC invasion and metastasis (62). Interestingly, soluble factors in adipose tissue MSCs were found to contribute to the proliferation and invasion of canine LC cells (63).

MSCs could inhibit the growth of LC cells and provide a targeted effect for the treatment of LC. NK4 modified MSCs could inhibit the growth and migration of MHCC-97H cells and tumor angiogenesis (64). *In vitro*, adipose tissue-derived MSCs can inhibit the growth of Huh7 HCC cells through IFN β-mediated JAK/STAT1 pathways (65). Similarly, UCMSCs could inhibit the growth of HepG2 cells and promote their apoptosis by down-regulating AFP, Bcl-2 and Survivin (66). To reduce the side effects of IFN-α in the clinical treatment of HCC, Su et al. found that IFN-α2b could be stably expressed by BMSCs, thereby inhibiting the progression of LC cells by reducing Notch1 expressions (67). In addition, Xie et al. discovered that IFN-β gene-modified BMSCs could stably secrete high levels of IFN-β, and then inhibit HCC through inhibiting the AKT/FOXO3a pathway. These results showed that BMSC/INF-α2b and BMSC/INF-β could be used as effective treatment strategies for LC (68). By down-regulating the Wnt/β-catenin signaling pathway, MSC-HNF4α was also suggested to reduce the growth and metastasis of LC (69). The co-expression of IFN-γ and IL-10 in BMSCs was confirmed to inhibit HCC by modulating cell cycle regulators and MAPK pathways (70). The Wnt signaling pathway was found to function in MSC-mediated LC cell suppression. MSCs-secreted Dkk-1 could inhibit the expression of Wnt signaling pathway-related factors in tumor cells, including Bcl-2, c-Myc, β-catenin and Survivin. Therefore, the absence of Wnt signaling pathway related factors may inhibit the proliferation of HepG2 cells and promote their apoptosis (71, 72). The miR-20a-3p carried by osteogenic MSC-derived extracellular vesicles could target c-FLIP and increase the level of TRAIL in HCC cells, thereby promoting TRAIL-related cell apoptosis (73). Moreover, Li et al. demonstrated that the BMSC-derived exosome miR-338-3p could inhibit the progression of HCC by targeting ETS1, which may provide a promising therapeutic target for HCC (74). Similarly, several studies have found that sorafini and MSCs have a synergistic anti-tumor effect on LC cells. The combination therapy of sorafini and MSCs can be used as a new treatment option for a transplant model for HCC (75, 76). Interestingly, Groth et al. investigated that porcine MSCs have a certain immune tolerance and are greatly similar to hMSCs. Thus, they believed that the potential of porcine MSCs for heterogeneous use in the treatment of LC is also prospective (77).

MSCs deserve attention in the recurrence of LC. To evaluate the potential of the stTRAIL (secretable form of TRAIL) gene therapy in the treatment of radiofrequency ablation in HCC, Deng et al. found that rat BMSCs could provide a stable source of stTRAIL, which may be suitable for recurrence of HCC in patients after receiving radiofrequency ablation (78). To remove postoperative residues of HCC and avoid the metastasis, Yuan et al. developed a conditioned replication adenovirus that could be loaded on hUCMSCs. They also found that MSCs could replicate adenoviruses to specifically eliminate HCC cells. The targeted therapy strategy is a hopeful approach to solving the problem of postoperative residual and metastasis of HCC (79). hMSCs were found to inhibit metastasis in HCC models and could be used to control metastatic recurrence of HCC. However, Li et al. found in mouse models that the presence of MSCs in the host may cause a recurrence of HCC (80). Nowadays, MSCs are rarely studied on the recurrence of LC, and the specific role and its mediating mechanism remain unclear. More studies are needed to support the role and mechanism of MSCs in LC recurrence.

The roles of MSCs in the metastasis, inhibition and treatment of LC are of great importance. And MSCs also play a certain role in the recurrence of LC. An increasing number of studies showed that UCMSCs, hMSCs and BMSCs could achieve metastasis, progression and inhibition of LC by directly acting or through their secretions and derivatives, which may provide guidance for the practical applications of MSCs in LC.

## Roles of Mesenchymal Stem Cells in Colorectal Cancer

CRC is a common malignant tumor in the GI tract. Its morbidity and mortality rate are second only to GC, EC and primary LC among digestive system malignancies (81). In recent years, it has been implemented that MSCs are closely related to the occurrence and development of CRC, which could guide a potential direction for the diagnosis and diagnosis of CRC.

It was found that MSCs could promote the progression of CRC. MSCs were proved to promote the progression of CRC by activating AMPK/mTOR-mediated NF-κB (82). Through IL-6/JAK2/STAT3 signal transduction, human CRC MSCs could increase the migration and invasion of CRC (83). Of note, rat BMSCs were found to promote the migration and invasion of CRC (84). Oh et al. demonstrated that after co-culturing MSCs with CRC cells, the TGF-β1 and p53 in MSCs increased, which enhanced the invasion and proliferation of CRC (85). hMSCs could differentiate into cancer-associated fibroblasts through CXCR4/TGF-β1 signaling, thereby promoting the growth and metastasis of CRC (86). Aging MSCs could stimulate the growth of CRC cells by expressing Galectin-3 (87). It was emphasized that BMSCs could promote the development of CRC through CCR5 (88). BMSCs could also promote CRC progression *via* signaling paracrine neuregulin 1/HER3 (89). Wang et al. also found that the IL-8 secreted by BMSCs could promote the angiogenesis and growth of CRC (90). Additionally, IL-6 secreted by MSCs could also signal through STAT3 to increase the number of colorectal initiating tumor cells and promote tumor formation (91).

Inversely, MSCs have been proved to inhibit the progression of CRC. BMSCs containing exosome microRNA-16-5p can inhibit the proliferation, migration and invasion of CRC by down-regulating ITGA2, then limiting the progression of CRC (92). hMSCs derived from pregnancy tissues are able to inhibit the proliferation of CRC cells by using different combinations of bioactive molecules (93). Tang et al. found that MSCs can migrate to colonic tissue and induce Treg cell differentiation through Smad2, thereby inhibiting the development of colitis-related CRC (94). Similarly, He et al. found that BMSCs could migrate to the colon, inhibit chronic inflammation and regulate the imbalance of the intestinal microbiota, thereby inhibit the development of CRC (95). Additionally, MSCs could inhibit the growth of CRC by modulating the immune components in the colorectal tumor microenvironment (96). Placental-derived MSCs expressing endostatin were revealed to have an inhibitory effect on CRC (97). Luetzkendorf et al. demonstrated that hMSCs containing lentiviral TRAIL transgenes could inhibit the growth of CRC (98). Moreover, exosomes derived from MSCs were reported to have an inhibitory effect on CRC in many studies. Upregulating the exosome miR-3940-5p derived from MSCs could inhibit the EMT and invasion of CRC cells and inhibit the metastasis and growth of tumors *in vivo*. Meanwhile, MSC-exosome miR-3940-5p were suggested to directly bind to ITGA6, thereby promoting CRC invasion and tumor progression by up-regulating TGF-β1 signaling (99). Exosome miR-4461, derived from BMSCs, could inhibit the migration and invasion of HCT116 and SW480 cells by down-regulating the expression of COPB2 in CRC (100). Exosome miR-22-3p derived from MSCs were also revealed to inhibit the proliferation and invasion of CRC *via* the RAP2B and PI3K/AKT pathways (101). Exosome miR-424 from BMSCs could inhibit the malignant behavior of CRC cells by targeting TGFBR3 (102). Liu et al. found that miR-15a derived from MSCs could inhibit the immune escape of CRC cells by modulating the KDM4B/HOXC4/PD-L1 axis (103). In addition, Zhao et al. also discovered that microRNA-34a-5p derived from tumorigenesis of CRC could be inhibited by MSC-derived extracellular vesicles through the c-MYC/DNMT3a/PTEN axis (104).

It was reported to MSCs play an important role in the treatment of CRC. MSCs could induce tumor matrix formation and EMT through the expression of SPARC in CRC, suggesting that SPARC in CRC cells may be used as a new target marker (105). IL7-IL12 engineered MSCs were reported to improve the attack of CAR-T cells on CRC cells, thereby improving the efficacy in the treatment of solid malignancies (106). Babaei et al. found that MSCs carrying oncolytic reovirus would enhance the anti-tumor activity of a mouse model of CRC (107). Additionally, it was showed that the combination of oncolytic virus delivered by MSCs and prodrug activation could inhibit tumor growth without causing toxicity to the host’s vital organs, which would improve the effectiveness and safety of CRC treatment and present a new method for the development of oncolytic virus therapies for cancers (108).

The role of MSCs in the growth, metastasis, inhibition and treatment of CRC has aroused more and more attention. In recent years, studies have revealed the mechanism by which MSCs act on CRC, which showed great potential of MSCs in the treatment of CRC. However, the involving studies are still insufficient, and researchers should perform more studies to investigate the clinical application of MSCs to CRC.

## Roles of Mesenchymal Stem Cells in Pancreatic Cancer

Pancreatic cancer is one of the common malignant tumors of the digestive tract (109). The five-year survival rate after diagnosis of pancreatic cancer is about 10%, indicating that patients with pancreatic cancer are likely to have the poor prognosis. The clinical symptoms of pancreatic cancer are insidious and atypical, so it is difficult to diagnose and cure clinically (110). In recent years, MSCs have been thought to function in the growth and metastasis of pancreatic cancer. And MSCs could inhibit the progression of pancreatic cancer, which may extend options for the treatment of pancreatic cancer.

MSCs could promote the progression of pancreatic cancer. It was found that cellular interactions between cancer cells and MSCs in the matrix of pancreatic ductal adenocarcinoma could regulate specific secretory molecules, thus promoting the progression of pancreatic cancer (111). Kabashima et al. found that MSC-derived myofibroblasts could regulate the EMT of pancreatic cancer cells through intermediate Notch signals, and have the function of maintaining the stem cell-like characteristics of tumor initiation (112). Moreover, MSCs were reported to promote the growth of pancreatic tumors by inducing alternating polarization of macrophages (113). MSCs expressing VEGF were also considered to promote pancreatic cancer angiogenesis (114). Ding et al. reported that exosomes secreted by hUCMSCs would accelerate the growth of pancreatic ductal adenocarcinoma by transferring miR-100-5p (115).

MSCs were reported to be closely related to the inhibition in the pancreatic cancer progression. Studies have shown that MSCs would exhibit an inherent inhibitory effect on pancreatic cancer cells (116). Similarly, Mohr et al. found that the combination of systemic MSC-mediated delivery of soluble TRAIL and XIAP inhibition could inhibit the metastatic growth of pancreatic cancer (117). IL10-modified hMSCs could also inhibit the growth of pancreatic cancer by inhibiting the secretion of pro-inflammatory cytokines IL6 and TNF-α and tumor angiogenesis (118). BMSCs could strongly inhibit the proliferation and migration of the pancreatic cancer cell line (119). MSC-IL15 were also improved to significantly inhibit the growth of pancreatic tumors. In addition, Jing et al. found that after MSC-IL15 treatment, the recovered mice were resistant to pancreatic tumor re-attack, suggesting that MSC-IL15 induced a tumor-specific T cell immune memory response, thereby eradicating established pancreatic tumors in mice (120). By activating the CCL5 promoter, MSCs would actively home to the primary pancreatic tumor matrix. Moreover, in the presence of the drug ganciclovir, HSV-TK transfected MSCs could significantly reduce the incidence of growth and metastasis of primary pancreatic tumors (121). MSC-derived exosomes also function in inhibiting pancreatic cancer. Song et al. found that exosome miRNA-1231 derived from BMSCs could inhibit the activity of pancreatic cancer (122). Similarly, Wu et al. found that BMSCs carrying the exosome miR-126-3p could inhibit the proliferation, invasion and metastasis of pancreatic cancer by targeting ADAM9, thereby promoting apoptosis of pancreatic cancer (123). hMSC-derived exosomes miR-143 could promote pancreatic cancer cell apoptosis and inhibit cell growth and invasion through the regulation of target genes (124). hUCMSCs carrying hsa-miRNA-128-3p could also inhibit pancreatic ductal cell carcinoma by inhibiting Galectin-3 (125). These above studies all suggested that MSC-derived exosomes could serve as potential targets for the treatment of pancreatic cancer.

MSCs may provide novel strategies for the treatment of pancreatic cancer. It was implemented that exosomes derived from BMSCs would have excellent penetration, matrix resistance and overcome chemical resistance, providing a prospective approach for targeted therapy for pancreatic cancer (126). Miyazaki et al. reported that adipose MSCs could differentiate into different pancreatic cancer-associated fibroblast subtypes, thereby driving tumor heterogeneity and playing a major role in the progression and drug resistance of pancreatic ductal adenocarcinoma (PDAC) (127). MSCs could increase the expression of SNHG7 in pancreatic cancer cells, which would promote the dryness and Folfirinox resistance of pancreatic cancer cells through the Notch1/Jagged1/Hes-1 signaling pathway (128). By preloading light-expressing myxoma virus into adipose-derived MSCs and intraperitoneal administration, oncolytic viruses could be efficiently delivered to the PDAC site and mediate improved tumor regression (129). hMSCs could be used for gene therapy of pancreatic cancer by using non-viral vectors to deliver TRAIL genes. Han et al. also found that the use of photochemical internalization could improve the transfection efficiency of TRAIL secreted from hMSCs and the tumor homing properties of hMSCs (130). Moreover, Kallifatidis et al. revealed that lentivirus richly expressing enhanced green fluorescent protein could transfer genes to MSCs. They also found a purely transduced MSC population could be quickly obtained after puromycin selection, which would improve the prospects of MSCs as gene therapy vectors to treat pancreatic cancer (131). Schug et al. also found that a therapeutic strategy consisting of MSC-mediated NIS gene delivery images and ^131^I application could also lead to a significant delay and reduction in the growth of pancreatic cancer (132).

The roles of MSCs in the metastasis, inhibition and treatment of pancreatic cancer cells have been emphasized. An increasing number of studies showed that MSCs could directly or indirectly achieve metastasis, progression and inhibition of pancreatic cancer cells. More efforts should be made to achieve more advance in the clinical use of MSCs in terms of pancreatic cancer.

## Conclusion and Prospects

**Figure 1:**
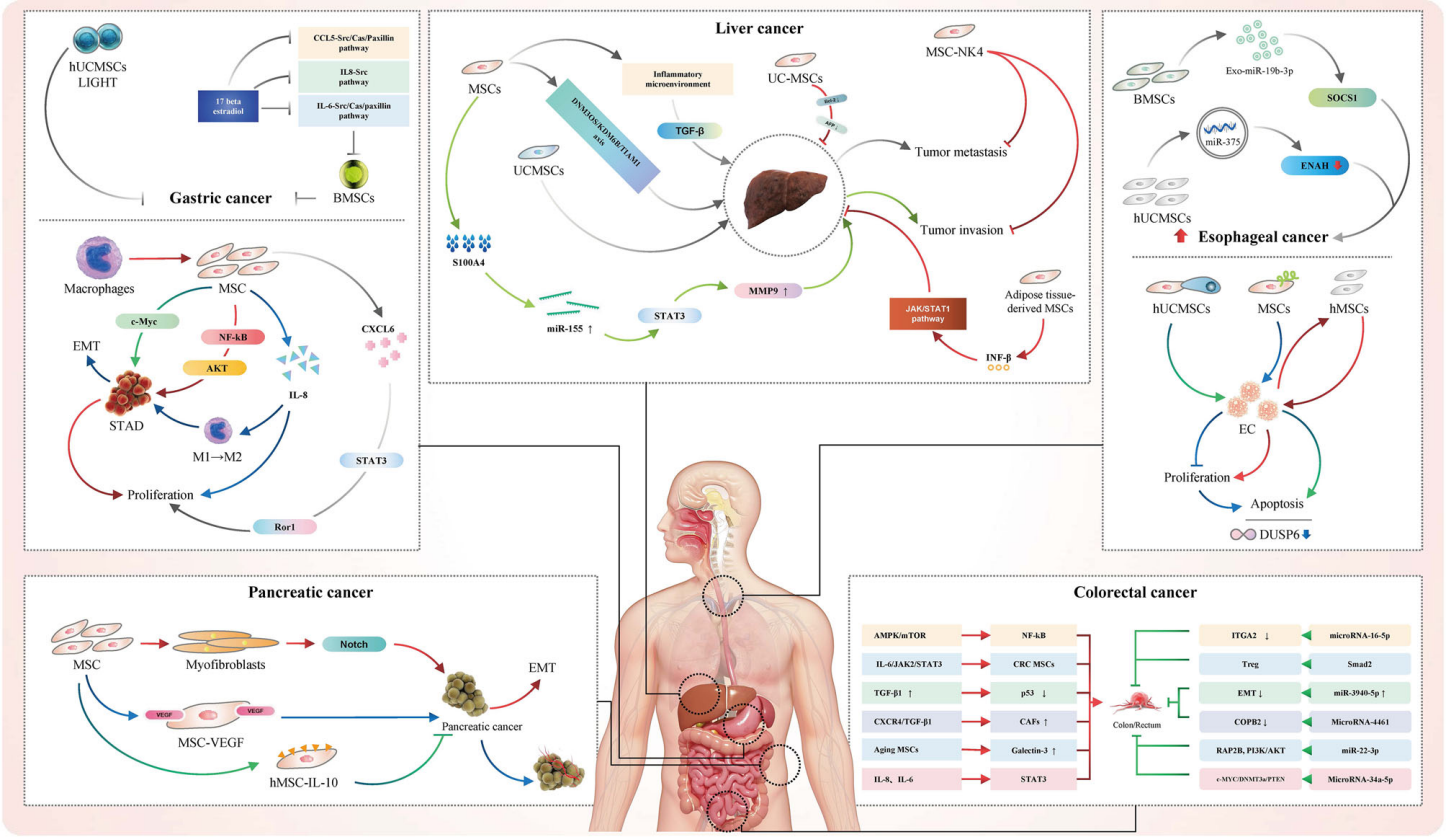
The roles of MSCs in different GI cancers.

To summary, MSCs pose a two-way effect on GI cancers. It could promote the metastasis and growth of tumor cells, and to the contrary, it would also inhibit the progression of tumor cells. It was clear that different MSCs may have different effects on GI cancers. Interestingly, the same MSCs may also have different effects on GI cancers through different pathways. Soluble factors and derived exosomes released by MSCs were thought to promote or inhibit GI cancers, providing potential targets for the treatment of GI cancers and as markers for prognostic testing. Of note, MSCs could be applied as important carriers for delivering anti-cancer biologics. Besides, it was reported that MSCs could act on the resistance of GI cancers (**Figure 1**).

It is worth noting that MSCs also have common roles in various GI cancers. In terms of the tumor-promoting and anti-tumor effects of MSCs, it was found that the effect of MSCs on GI cancers is mainly achieved through signal transduction pathways, of which Wnt signaling pathway is the most common in GC and LC. It was also concluded that interleukin secreted by MSCs matters in different GI cancers. GC-MSCs that massively secrete IL-6 and IL-8 could promote the growth of GC. IL-6 and IL-8 secreted by BMSCs could promote HCC metastasis and CRC growth, respectively. IL-10 modified hMSCs could inhibit the growth of pancreatic cancer, and IL-15 produced by MSCs could also significantly inhibit the growth of pancreatic cancer. TRAIL-mediated MSCs have inhibitory effects on EC, CRC and pancreatic cancer. Moreover, exosomes and their derivatives could also mediate the occurrence and development of GI cancers with the help of miRs.

So far, although studies on MSCs have made great progress, the researches on the prediction and treatment of GI cancers by MSCs are not enough. In addition, most studies on the treatment of GI cancer by MSCs still remain at the stage of cell and animal experiments, and the precise molecular mechanism of MSCs on GI cancers needs to be further investigated. More efforts should be made to explore the possibility of the clinical practice of MSCs in GI cancers.

## Author Contributions

ZX, MH, and JW had the idea for the article. ZH and CZ performed the literature search and data analysis. GZ and JW drafted and critically revised the work. All authors contributed to the article and approved the submitted version.

## Conflict of Interest

The authors declare that the research was conducted in the absence of any commercial or financial relationships that could be construed as a potential conflict of interest.

## Publisher’s Note

All claims expressed in this article are solely those of the authors and do not necessarily represent those of their affiliated organizations, or those of the publisher, the editors and the reviewers. Any product that may be evaluated in this article, or claim that may be made by its manufacturer, is not guaranteed or endorsed by the publisher.

## Abbreviations

MSCs, Mesenchymal stem cells; GI cancers, Gastrointestinal cancers; EC, Esophageal cancer; GC, Gastric cancer; LC, Liver cancer; CRC, Colorectal cancer; hUCMSCs, Human umbilical cord mesenchymal stem cells; hMSCs, Human mesenchymal stem cells; miRs, MicroRNAs; BMSCs, Bone marrow mesenchymal stem cells; EMT, Epithelial mesenchymal transformation; PD-L1, Programmed cell death ligand 1; HCC, Hepatocellular carcinoma; UCMSCs, Umbilical cord mesenchymal stem cells; stTRAIL, Secretable form of TRAIL; PDAC, Pancreatic ductal adenocarcinoma.
